# Autotransporter domain-dependent enzymatic analysis of a novel extremely thermostable carboxylesterase with high biodegradability towards pyrethroid pesticides

**DOI:** 10.1038/s41598-017-03561-8

**Published:** 2017-06-14

**Authors:** Xianghai Cai, Wei Wang, Lin Lin, Dannong He, Gang Huang, Yaling Shen, Wei Wei, Dongzhi Wei

**Affiliations:** 1 State Key Laboratory of Bioreactor Engineering, Newworld Institute of Biotechnology, East China University of Science and Technology, Shanghai, 200237 People’s Republic of China; 2 Research Laboratory for Functional Nanomaterial, National Engineering Research Center for Nanotechnology, Shanghai, 200241 People’s Republic of China; 3 Shanghai University of Medicine and Health Sciences, Shanghai, 200093 People’s Republic of China

## Abstract

The *Est*PS1 gene, which encodes a novel carboxylesterase of *Pseudomonas synxantha* PS1 isolated from oil well-produced water, was cloned and sequenced. *Est*PS1 has an open reading frame of 1923 bp and encodes the 640-amino acid carboxylesterase (EstPS1), which contains an autotransporter (AT) domain (357–640 amino acids). Homology analysis revealed that EstPS1 shared the highest identity (88%) with EstA from *Pseudomonas fluorescens* A506 (NCBI database) and belonged to the carboxylesterase family (EC 3.1.1.1). The optimum pH and temperature of recombinant EstPS1 were found to be 8.0 and 60 °C, respectively. EstPS1 showed high thermostability, and the half-lives (T_1/2_ thermal inactivation) at 60, 70, 80, 90, and 100 °C were 14 h, 2 h, 31 min, 10 min, and 1 min, respectively. To understand the role of the AT domain in carboxylesterase, AT domain-truncated carboxylesterase (EstPS1**Δ**AT) was generated. EstPS1**Δ**AT showed a clearly decreased secretion rate, owing to the AT domain strongly improved secretory expression in the heterogeneous system. EstPS1 degraded various pyrethroid pesticides, and hydrolysis efficiencies were dependent on the pyrethroid molecular structure. EstPS1 degraded all the tested pyrethroid pesticides and hydrolysed the *p*-nitrophenyl esters of medium-short-chain fatty acids, indicating that EstPS1 is an esterase with broad specificity.

## Introduction

Carboxylesterases (EC 3.1.1.1) are members of the α/β hydrolase family, which have been found in animals, plants, and microorganisms. Enzymes of the carboxylesterase family have a preference for carboxylate esters with short chains (<10 carbon atoms) as substrates, and they exhibit high thermostability, have a broad substrate specificity, demonstrate high regio/stereo-specificity, require no cofactor, and tolerate organic solvents^1^. Esterases and lipases from microorganisms have attracted attention for their potential applications in the hydrolysis and synthesis of important ester compounds for the pharmaceutical, food, biochemical, and biological interests^2, 3^. The carboxylesterase (rPPE) of *Pseudomonas putida* has been cloned, and its substrate binding mechanism was determined^4^. The carboxylesterase of *Pseudomonas citronellolis* ATCC 13674 has also been characterised^5^. Furthermore, the carboxylesterase of *Pseudomonas aeruginosa* PAO1 has been isolated and expressed in *Escherichia coli* ^6^. The combined structural, biochemical, and computational characteristics of carboxylesterase PA3859 from *P. aeruginosa* have also been examined^7^. Although many microbial carboxylesterases have been reported, there is limited information on carboxylesterases from *Pseudomonas* sp. Moreover, there are no studies on the carboxylesterase/lipase of *Pseudomonas synxantha* with regard to wild-type strain lipase activity, lipase gene cloning from *P. synxantha*, and carboxylesterases of recombinant strains. Thermostability is an important enzymatic property for commercial enzymes, and an increased reaction temperature would speed up the rates of enzyme-catalysed reactions or enhance substrate solubility, which can promote a faster transesterification reaction. Cloning of novel carboxylesterase genes with distinct features such as cold adaptability, thermostability, or catalytic ability is of interest for industrial applications^8, 9^.

Carboxylesterase is an intracellular serine hydrolase that belongs to the lipase GDSL-2 family. Members of the GDSL family possess the GDSX motif (equivalent to the classical GXSXG motif of lipases/esterases), in which nucleophilic Ser is located in block I of the protein^10^. The EstA of *P. aeruginosa* is an autotransporter (AT) that exhibits lipolytic activity, and its passenger domain is a member of the GDSL family of lipases. GDSL ATs are large proteins produced and secreted by gram-negative bacteria. They share a common architecture, including a signal peptide, passenger domain with a specific function, and translocation domain that forms a β-barrel and anchors the protein to the bacterial outer membrane^11^. ATs have various potential applications such as the surface display and extracellular expression of recombinant proteins. Indeed, ATs have been used as transport carriers for various heterologous fusion partners in *E. coli*; nevertheless, their physiological function remains unknown^12^.

Carboxylesterases can efficiently hydrolyse pyrethroids to their corresponding acids and alcohols to reduce toxicity^13^. Pyrethroid insecticides are synthetic analogues of the naturally occurring pyrethrum flowers and exhibit a high degree of hydrophobicity. Increased pyrethroid usage has resulted in the need to monitor environmental samples for the presence and potential toxic effects of pyrethroids. Carboxylesterases can rapidly degrade both type I and type II pyrethroids. These types of enzymes have been found to be effective for reducing pyrethroid-associated toxicity in both mammals and insects. Pyrethroids are much more toxic to aquatic organisms than to mammals. Laboratory tests have shown that pyrethroids are extremely toxic to fish such as the fathead minnow, rainbow trout, brook trout, bluegill, and sheepshead minnow^14^. Most studies have focused on the isolation of synthetic pyrethroids from pyrethroid-degrading microorganisms, such as beta-cyfluthrin from *Pseudomonas stutzeri* strain S1^15^, organophosphates and pyrethroids from *P. putida* KT2440^16^, and beta-cypermethrin from *P. aeruginosa* CH7^17^. These enzymes play a significant role in the metabolism and subsequent detoxification of many agrochemicals and pharmaceuticals. Therefore, it is important to identify the pesticide-degrading esterase in *Pseudomonas* species.

In a previous study, *P. synxantha* was isolated from oil well-produced water (Shengli Oilfield, Shandong Province, China) and characterised by morphological, physiological, biochemical, and 16 S rDNA sequence analysis^18^. The study identified *P. synxantha* as a gram-positive mesophilic bacterium, which displayed hydrolytic activity towards tributyrin with a large hydrolysis circle. In this study, we expressed and characterised an extremely thermostable carboxylesterase from *P. synxantha* PS1 and carboxylesterases from recombinant strains. Furthermore, a novel AT domain in the carboxylesterase of *P. synxantha* PS1 was identified, and the role of the AT domain in the activity, secretion, and thermostability of the enzyme was also determined using an AT domain-truncated enzyme. The AT domain could promote recombinant enzyme secretion into the extracellular space, which agrees with the results of previous studies. Additionally, the ability of recombinant carboxylesterase to hydrolyse pyrethroids was preliminarily evaluated. The enzyme catalysed the hydrolysis of short-chain phenyl acyl esters and pyrethroids. Owing to enzymatic properties such as high thermostability, high catalytic activity towards pyrethroid, and high tolerance towards polar solvents, this carboxylesterase has potential value in industrial applications, particularly for environmental protection.

## Results

### Gene library screening and gene analysis of thermostable carboxylesterase

Strain PS1 was identified as *P. synxantha* by 16S rDNA phylogenetic analysis and morphology evaluation. Based on genomic DNA library screening and sequencing data analysis, the thermostable esterase gene *Est*PS1 (GenBank accession number: KT070707) was identified and cloned for further study. Through PCR amplification, a 1923-bp DNA fragment encoding a polypeptide of 640 amino acids was cloned and sequenced. Sequence analysis revealed that the ORF start codon of the *Est*PS1 gene was ATG, and the G+C content (%) of *Est*PS1 was 46.7 mole %.

**Figure 1:**
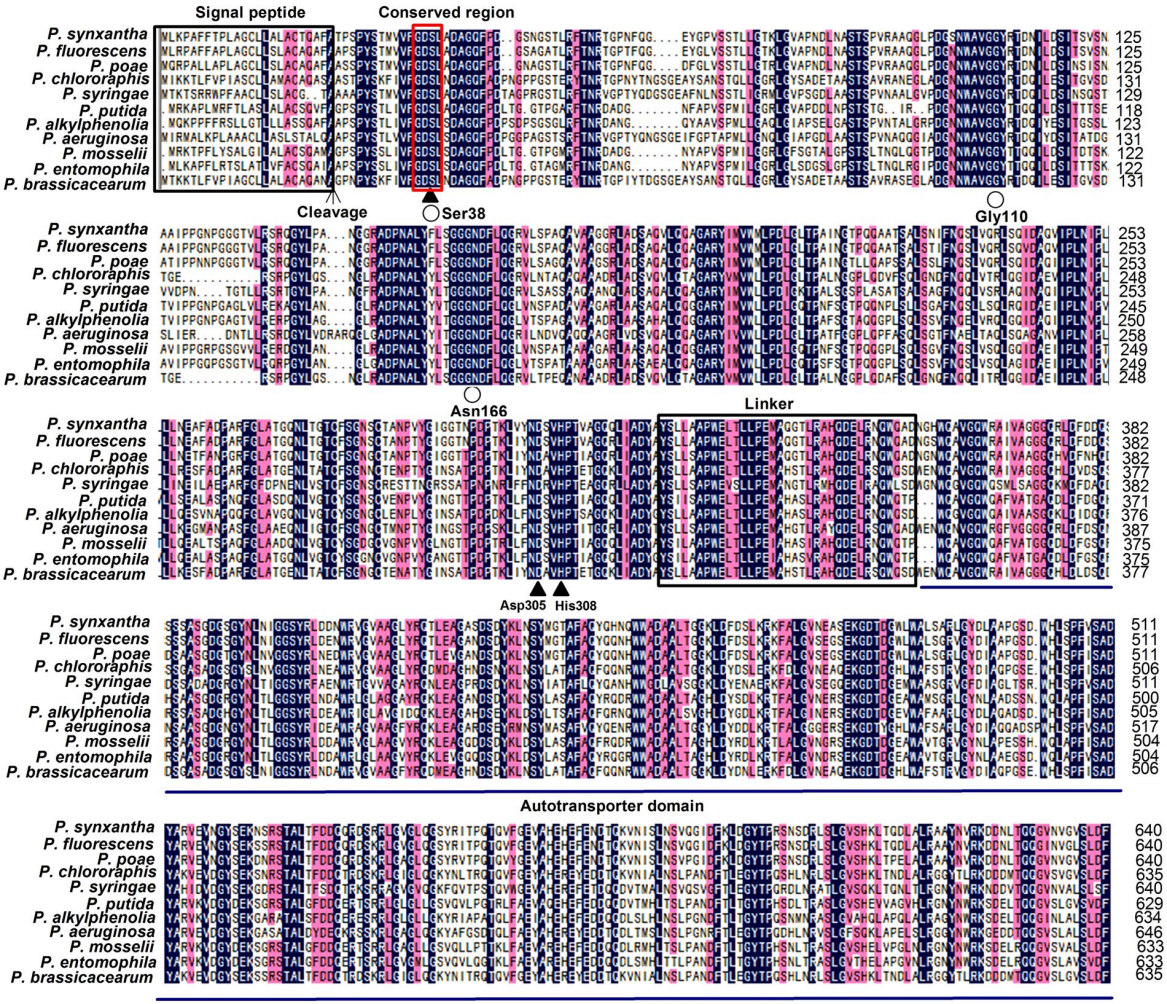
Conserved sequence alignment of EstPS1. The structures are denoted as follows: filled triangle, the catalytic site (Ser^38^, Asp^305^, and His^308^); empty circle, the putative oxyanion hole (Ser^38^-Gly^110^-Asn^166^). The reserved amino acid motif GDSL is boxed by the red line. The signal peptide and linker are also boxed. The consensus amino acid residues of the autotransporter domain are shown in underscore characters.

Homology analysis revealed that the EstPS1 of *P. synxantha* PS1 shared the highest identity (88%) with the carboxylesterase EstA of *Pseudomonas fluorescens* A506 (GI: 387159426), and it was 84% identical to the carboxylesterase of *Pseudomonas poae* RE1-1-14 (GI: 445198867) (76% to *Pseudomonas chlororaphi*s UFB2; GI: 829490642, 71% to *Pseudomonas syringae* UMAF0158; GI: 927283541, 69% to *P. putida* H8234; GI: 511519639, 70% to *Pseudomonas alkylphenolia* KL28; GI: 675318909, 69% to *P. aeruginosa* PA7; GI: 150958624, 70% to *Pseudomonas mosselii* SJ10; GI: 684194542, 70% to *Pseudomonas entomophila* L48; GI: 95101722, and 74% to *Pseudomonas brassicacearum* NFM421; GI: 327374765) (Fig. 1).

### Structural and conserved domain analysis of carboxylesterase

**Figure 2:**
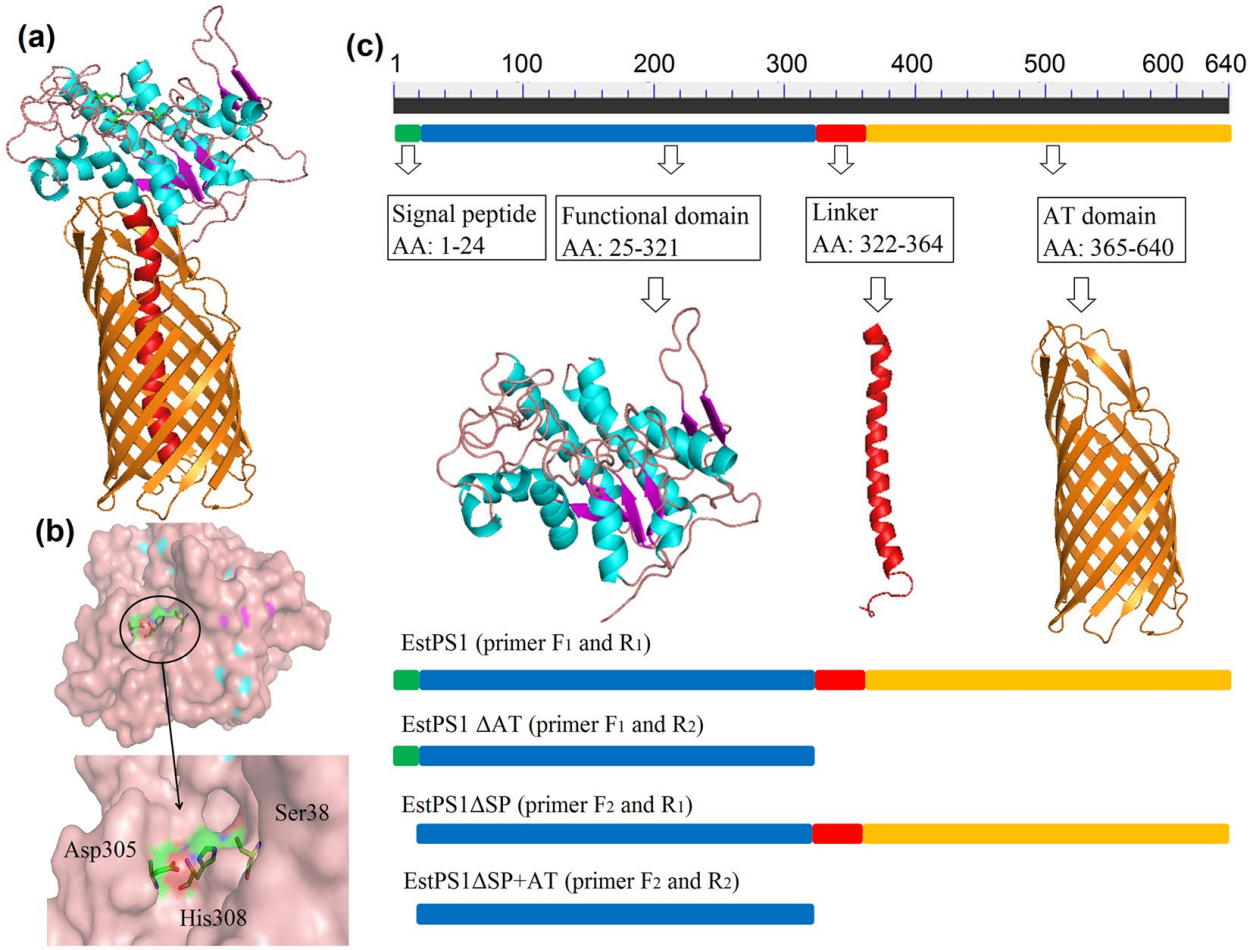
The protein model and conserved domain analysis. (**a**) 3D model of EstPS1. (**b**) The catalytic site (Ser^38^, Asp^305^, and His^308^) labelled in the 3D model. (**c**) Conserved domain analysis and AT domain expression strategy (EstPS1, EstPS1**Δ**AT, EstPS1**Δ**SP, and EstPS1**Δ**SP + AT).

EstPS1 was found to have a single catalytic domain of the alpha/beta hydrolase family and belong to the carboxylesterase family (EC 3.1.1.1). Sequence alignment of the conserved region (EstPS1) and other esterases from different bacterial sources was performed and revealed amino acid conservation in regions associated with catalysis and stabilization of the protein, e.g., the active site (Ser^38^), catalytic site (Ser^38^, Asp^305^, and His^308^), and putative oxyanion hole (Ser^38^-Gly^110^-Asn^166^). Given that this carboxylesterase has not been previously reported, the three-dimensional model of EstPS1 was predicted using the SWISS-MODEL server according to the crystal structure of EstA (PDB ID: 3KVN) from *Pseudomonas aeruginosa*, and the protein structure was viewed in PDB Viewer (Fig. 2a,b). The catalytic triad (Ser^38^, Asp^305^, and His^308^ residues) was observed in the regions. The conserved region, GDSL (a characteristic of carboxylesterase sequences from *Pseudomonas* sp.), is boxed. Additionally, the sequence of the signal peptide is shown in Fig. 1.

An AT domain (Gly^357^-Phe^640^) was identified in EstPS1 through enzyme domain analysis. This AT domain contained an N-terminal signal sequence that targets the enzyme to the Sec machinery in the inner membrane. Analysis of the signal peptide revealed a possible signal peptide of 24 amino acids in the N-terminal region; the peptide bond between the 24th and 25th amino acids (AFA-TP) was predicted to be cleaved by signal peptidase (SignalP Server)^19^. The molecular weight of EstPS1 was estimated as 68 kDa (34 kDa without the AT domain), and the pI value was calculated as 4.81 (4.75 without the signal peptide) by the ExPASy compute pI/Mw program algorithm (Table 1).

**Table 1:** Comparison between EstPS1 and EstPS1**Δ**AT.

Items	EstPS1	EstPS1**Δ**AT
Mw	68 kDa	34 kDa
pI	4.81	4.75
Secretion rate	9.7%	1.2%
Activity	2226 U/mg	973 U/mg
t_1/2_ (60 °C)	14 h	12 h
t_1/2_ (70 °C)	120 min	95 min
t_1/2_ (80 °C)	31 min	20 min
t_1/2_ (90 °C)	10 min	6 min
t_1/2_ (100 °C)	1 min	NM

### Expression and purification of recombinant EstPS1 with/without the AT domain

**Figure 3:**
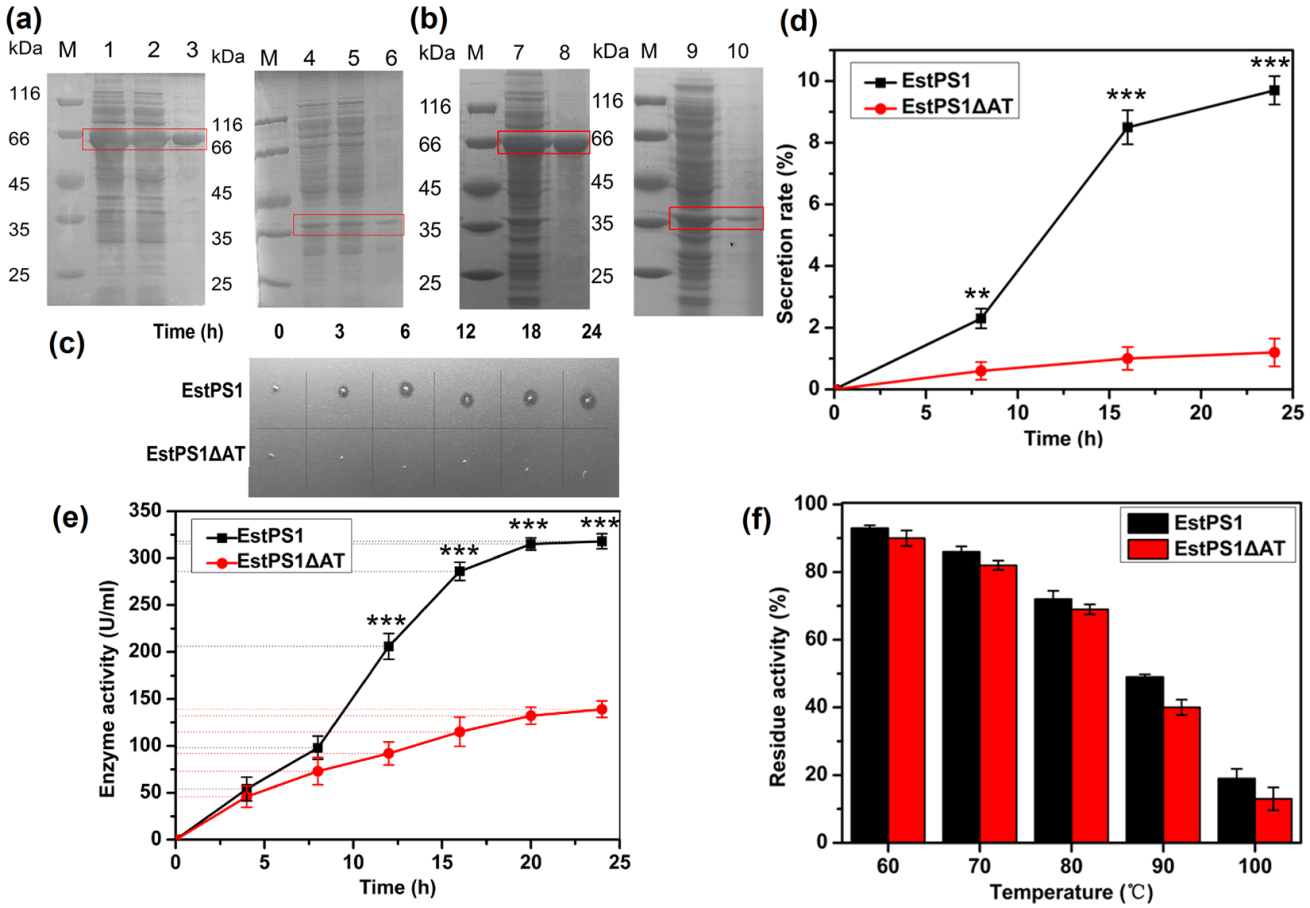
Role of the AT domain in recombinant EstPS1. (**a**) SDS-PAGE analysis of EstPS1 and EstPS1**Δ**AT. *lane M* - standard marker proteins; *lane 1,2* - supernatant of cell lysate (EstPS1); *lane 3* - purified protein of EstPS1; *lane 4,5* - supernatant of cell lysate (EstPS1**Δ**AT); *lane 6* - purified protein of EstPS1**Δ**AT. (**b**) SDS-PAGE analysis of EstPS1**Δ**SP and EstPS1**Δ**SP + AT. *lane M* - standard marker proteins; *lane 7* - crude extracts of EstPS1**Δ**SP; *lane 8* - purified protein of EstPS1**Δ**SP (inclusion bodies); *lane 9* - crude extracts of EstPS1**Δ**SP + AT; *lane 10* - purified protein of EstPS1**Δ**SP + AT (inclusion bodies). (**c**) Transparent zones of EstPS1 and EstPS1**Δ**AT in the solid induction medium at different hours. (**d**) Effect of the AT domain on secretion rate at 0 h, 8 h, 16 h, and 24 h. Secretion rate is defined as the enzyme activity of culture medium in proportion to the total activity. Data represent mean ± SD (n = 3); *p < 0.05, **p < 0.01, ***p < 0.001 by *Student’s-t* test. (**e**) Comparison of the total activity (fermentation broth) between EstPS1 and EstPS1**Δ**AT with respect to the AT domain. (**f**) Residual activities of the enzymes after incubation at various temperatures (60–100 °C) for 10 min.

The recombinant strains (BL21-*Est*PS1, BL21-*Est*PS1**Δ**AT, BL21-*Est*PS1**Δ**SP, and BL21-*Est*PS1**Δ**SP + AT; Fig. 2c) were grown to saturation in LB medium supplemented with the appropriate antibiotic to express the recombinant proteins. SDS-PAGE results showed that the recombinant proteins of *Est*PS1 and *Est*PS1**Δ**AT appeared mostly as soluble proteins under low-temperature induction (20 °C) **(**Fig. 3a **)**. Crude extracts and purified fractions SDS Page images of EstPS1**Δ**SP and EstPS1**Δ**SP+AT were shown in Fig. 3b. The optimal expression conditions were an IPTG concentration of 0.1 mM, induction temperature of 20 °C, and induction time of 20 h. Additionally, the recombinant proteins of *Est*PS1**Δ**SP and *Est*PS1**Δ**SP+AT were present in inclusion bodies, even under low-temperature induction. Through Ni-NTA purification procedures, the highest enzyme specific activities of purified EstPS1 and EstPS1**Δ**AT were 2226 and 973 U/mg, respectively (Table 1).

### Biochemical characterization of purified recombinant carboxylesterase

**Figure 4:**
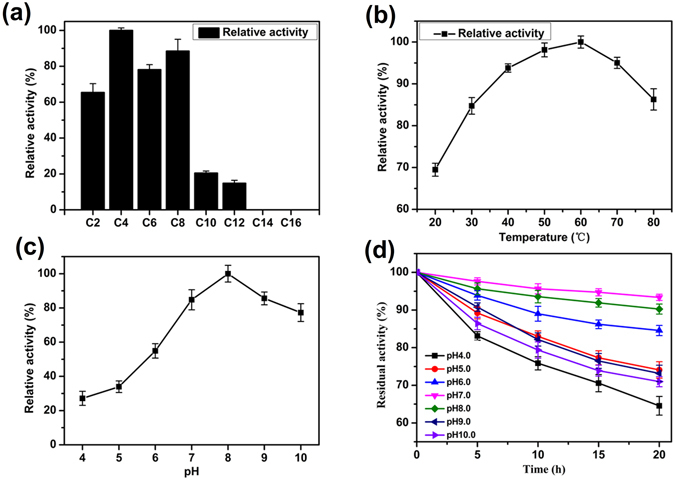
Characterization of recombinant EstPS1. (**a**) Determination of the optimum substrate for EstPS1. The relative activity (%) was obtained by comparison of the substrates (C4). (**b**) Optimum temperature of *p*-nitrophenyl butyrate for EstPS1 activity. (**c**) Optimum pH of *p*-nitrophenyl butyrate with EstPS1. (**d**) pH stability of *p*-nitrophenyl butyrate with EstPS1 in different buffers for 20 h at 30 °C.

The ability of the purified enzyme to hydrolyse various *p*-nitrophenyl ester (C2–C18) substrates was examined at 60 °C and pH 8.0. Among the various examined esters, EstPS1 showed the highest activity with *p*-nitrophenyl butyrate (2226 U/mg). The substrate specificity of EstPS1 toward the *p*-nitrophenyl esters of various fatty acids is shown in Fig. 4a. A comparison of the catalytic efficiency values (*kcat*/*Km*) of various substrates is shown in Table 2. The results indicated the dependence of these values on the aliphatic chain length of the substrate. Short-chain *p*-nitrophenyl esters appeared to be the preferred substrates, whereas *p*-nitrophenyl esters of long-chain fatty acids were poor substrates. EstPS1 showed no activity toward *p*-nitrophenyl myristate and *p*-nitrophenyl palmitate. The activity toward the *p*-nitrophenyl esters of medium-chain fatty acids was lower than that toward *p*-nitrophenyl butyrate. These results indicated that the purified enzyme (EstPS1) is an esterase and not a lipase.

**Table 2:** Substrate specificity of recombinant EstPS1.

Substrate	Specific activity (μmol min^−1^ mg^−1^)	*Km* (μM)	*kcat* (s^−1^)	*kcat/Km* (s^−1^ μM^−1^)
*p*-Nitrophenyl acetate	278 ± 21	102 ± 12	308 ± 23	3.02 ± 0.23
*p*-Nitrophenyl butyrate	425 ± 6	43 ± 8	481 + 7	11.19 ± 0.16
*p*-Nitrophenyl caproate	332 ± 12	76 ± 7	376 ± 14	4.95 ± 0.18
*p*-Nitrophenyl caprylate	376 ± 28	61 ± 10	417 ± 31	6.83 ± 0.51
*p*-Nitrophenyl decanoate	87 ± 5	362 ± 13	98 ± 6	0.271 ± 0.02
*p*-Nitrophenyl laurate	63 ± 7	391 ± 9	71 ± 8	0.182 ± 0.02
*p*-Nitrophenyl myristate	0	NM	NM	NM
*p*-Nitrophenyl palmitate	0	NM	NM	NM
Carbaryl	13.6 ± 0.86	0.21 ± 0.01	15.4 ± 0.98	73.3 ± 4.67
cis-Cypermethrin	6.5 ± 0.51	0.62 ± 0.031	7.4 ± 0.61	11.9 ± 0.98
trans-Cypermethrin	7.5 ± 0.58	0.53 ± 0.028	8.5 ± 0.66	16.1 ± 1.25
Fenpropathrin	12.8 ± 0.79	0.25 ± 0.014	14.5 ± 0.88	58 ± 3.52
Fenvalerate	4.3 ± 0.32	0.84 ± 0.042	4.9 ± 0.36	5.8 ± 0.43
Bifenthrin	0.92 ± 0.02	1.25 ± 0.061	1.1 ± 0.02	0.88 ± 0.02

The effect of pH on carboxylesterase activity was determined using *p*-nitrophenyl butyrate as a substrate at various pH levels at 60 °C. Purified EstPS1 exhibited higher esterase activities over a pH range of 6.0–9.0, among which the highest specific enzyme activity was observed at pH 8.0 (Fig. 4c). EstPS1 activity was markedly decreased below pH 5.0, and approximately 30% of the maximal activity was observed under this condition. The stability of the purified enzyme was investigated in buffer solutions over a pH range of 4.0–10.0. The results demonstrated the stability of lipase under alkaline conditions, and it was most stable at pH 6.0–9.0, with the highest stability at pH 7.0 (retaining 95% activity after 20 h) (Fig. 4d).

### Optimum temperature and thermal stability of recombinant carboxylesterase

The optimum temperature of purified EstPS1 was determined by assaying enzyme activity at different temperatures (20, 30, 40, 50, 60, 70, and 80 °C) in 50 mM Tris-HCl buffer (pH 8.0). Purified EstPS1 exhibited higher esterase activities over a temperature range of 40–70 °C (over 80% of the highest activity), with the highest specific enzyme activity (2226 U/mg) at 60 °C (Fig. 4b). Residual activities were determined under standard assay conditions. EstPS1 was very stable at 20–70 °C, and more than 50% of its activity was retained after 14 h at 60 °C. Even after incubation for 10 min at 100 °C, the enzyme retained 19% of the maximal activity. The EstPS1 half-lives at 60, 70, 80, 90, and 100 °C were 14 h, 2 h, 31 min, 10 min, and 1 min, respectively, indicating that the enzyme had relatively good thermostability.

### Effect of AT domain truncation on secretion expression and thermostability

Our results revealed that the AT domain enhanced the secretion of the recombinant enzymes, which was qualitatively observed in the solid induction medium (Fig. 3c). EstPS1 showed a larger transparent zone than the AT domain-truncated enzyme (EstPS1**Δ**AT), which increased over time. Additionally, the secretion rate was calculated based on the secreted enzyme activity (Fig. 3d **)**. The enzyme activity of EstPS1 and EstPS1**Δ**AT was 2226 and 973 U/mg, respectively (Table 1 **)**. EstPS1**Δ**AT had a secretion rate of 1.2%, whereas EstPS1 had a rate of 9.7%. Initially, their values were relatively similar; however, the differences increased over time. Therefore, the AT domain facilitated enzyme release from the cell. Total enzyme activity was also influenced by the AT domain (Fig. 3e). Recombinant EstPS1 and EstPS1**Δ**AT had a similar optimal pH (8.0) and temperature (60 °C), whereas the thermostability of EstPS1 was higher than that of EstPS1**Δ**AT (Fig. 3f). A comparison of the thermal stability between EstPS1 and EstPS1**Δ**AT is shown in Table 1.

### Pesticide specificity

**Figure 5:**
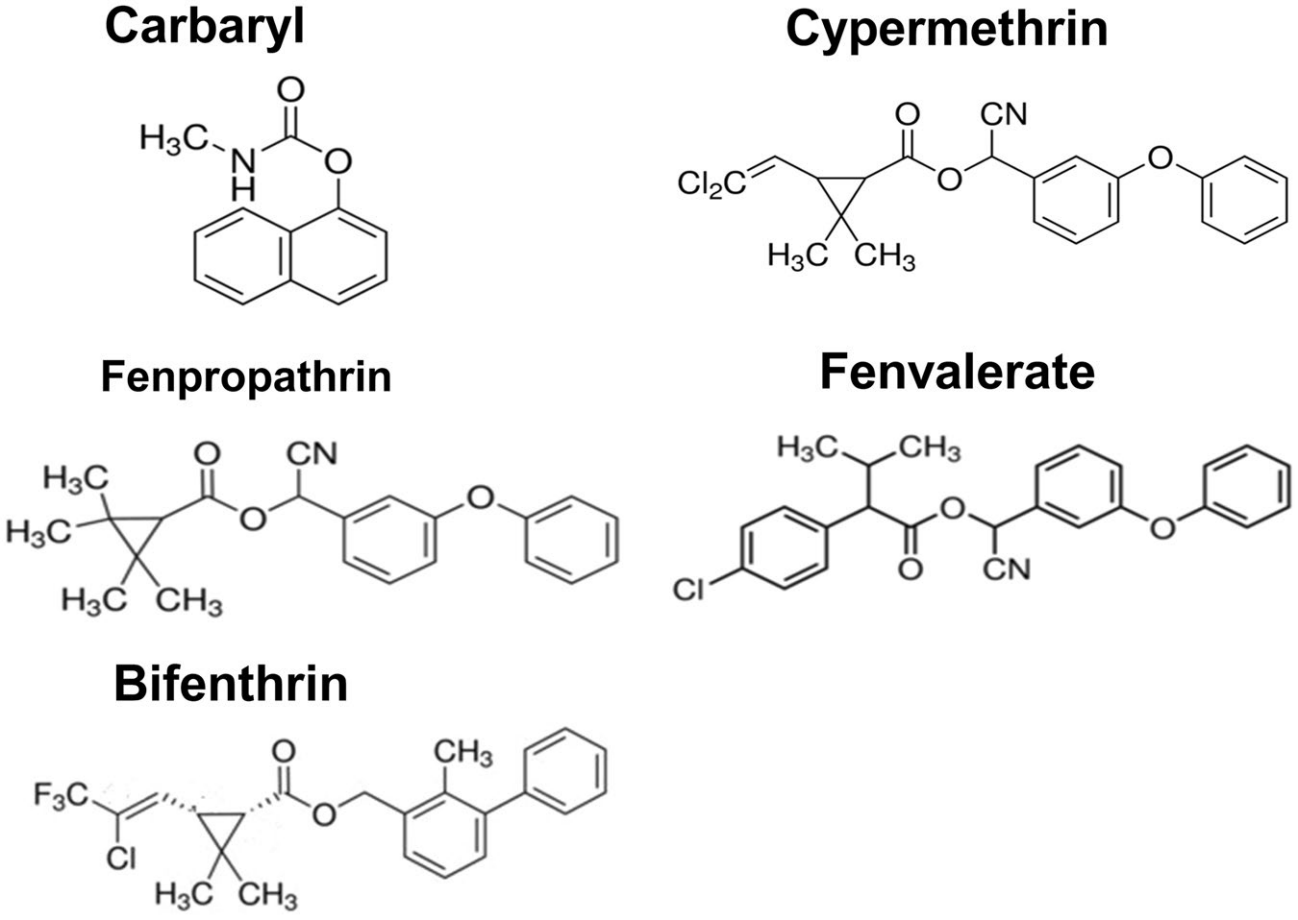
Structures of the tested pesticides (carbaryl, *cis*-cypermethrin, *trans*-cypermethrin, fenpropathrin, fenvalerate, and bifenthrin).

Various pesticides were tested for substrate specificity with recombinant EstPS1 (Fig. 5). As shown in Table 2, EstPS1 hydrolysed the pesticides at different hydrolysis rates in the following decreasing order: carbaryl > fenpropathrin > *trans*-cypermethrin > *cis-*cypermethrin > fenvalerate > bifenthrin. Carbaryl was the most rapidly hydrolysed pesticide with a specific activity of 13.6 ± 0.86 μmol min^−1^ mg^−1^; on the other hand, bifenthrin demonstrated the slowest rate, and *cis*-cypermethrin was hydrolysed at a rate approximately equal to that of *trans*-cypermethrin. Therefore, the pyrethroid-hydrolysing esterase was capable of hydrolysing a relatively wide range of compounds with similar chemical linkages at normal temperatures, suggesting that it has a broad substrate specificity. This feature was similar to that of the pyrethroid hydrolases; however, EstPS1 displayed a higher hydrolysis rate^14, 20, 21^. The *Km* and *kcat* values were calculated by fitting the data to the Michaelis-Menten equation. The purified enzyme had different *Km* values ranging from 0.21 to 1.25 μM with the various tested pesticides as substrates.

### Nucleotide sequence accession numbers

The nucleotide sequences of the 16 S rDNA and *Est*PS1 genes of *P. synxantha* strain PS1 were deposited in the GenBank database under accession numbers KM232508 and KT070707, respectively.

## Discussion

*Pseudomonas* species are well known for their ability to produce and secrete a large number of useful extracellular enzymes. *Pseudomonas* lipolytic enzymes have many advantages compared with other bacterial lipases^22^, including temperature stability and organic solvent tolerability. In addition, the enzymes have potential applications in many hydrolysis and synthesis catalytic reactions, particularly in non-aqueous biocatalysis. Cloning of the novel lipase gene with distinct features (thermal tolerance, organic solvent tolerability, and catalytic reaction) is of interest for industrial applications^23, 24^. Although many lipases (including carboxylesterases) have been reported, there are no reports of *P. synxantha* carboxylesterase/lipase. Limited studies have examined *P. synxantha* (referred to as the wild strain), and there are no studies on recombinant strains. Here, we characterised a new lipolytic enzyme (EstPS1) cloned from a *P. synxantha* strain referred to as PS1. Homology analysis revealed that EstPS1 from *P. synxantha* shared 60–88% identity with esterases from *Pseudomonas* sp. The sequence, structure, and properties of EstPS1 were compared with those of esterases identified in the classification of Arpigny and Jaeger, and EstPS1 was assigned to family II (I–VIII)^1^. This family shares a conserved motif domain, GDSL. Sequence alignment with putative esterases of *Pseudomonas* sp. indicated that the enzyme contained the same catalytic triad, consisting of Ser^38^, Asp^305^, and His^308^, and a GDSL structural motif. The molecular mass of EstPS1 was approximately 68 kDa, which is similar to that of other pyrethroid-hydrolysing enzymes, such as EstP (73 kDa) from *Klebsiella* sp. strain ZD112^25^, pyrethroid-hydrolysing carboxylesterase (60 kDa) from mouse liver microsomes^26^, carboxylesterase E3 (58.6 kDa) from *N. cincticeps* Uhler^27^, and pyrethroid hydrolase (56 kDa) from *A. niger* ZD11^28^.

ATs have been widely used in biotechnological applications, such as the surface display and secretion of heterologous proteins^12, 29, 30^. Recent studies have shown that passenger domains are secreted through the outer membrane in a C-to-N-terminal order. Despite their importance in biotechnological applications, the role of the transporter domain and the mechanism of passenger translocation across the bacterial outer membrane are not well understood. ATs with GDSL passenger domains (contain an N-terminal signal sequence) target the proteins to the Sec machinery in the inner membrane. Through this mechanism, the proteins are secreted into the periplasm where their signal peptides are cleaved by a signal peptidase^11^. The signal peptide region reportedly contributes to enzyme activity and stability in esterases of the GDSL family, which was confirmed by our results. In this study, EstPS1 with an added signal peptide region was soluble, whereas EstPS1**Δ**SP was insoluble. To study the function of the AT domain, the secretion rates of EstPS1 and EstPS1**Δ**AT were determined under the same conditions. The AT domain promoted recombinant enzyme secretion into the extracellular environment, which agrees with the results of previous studies^12, 31^. In our study, both the secretion rate and thermostability was enhanced by the AT domain. The rigid structure of the AT domain may have stabilised the recombinant enzyme structure.

Homology results revealed distinct properties of the novel recombinant enzyme with respect to enzyme characteristics (particularly high thermal tolerance) and kinetic parameters. The optimum temperature (60 °C) of the recombinant enzyme is similar to that of carboxylesterase from *P. aeruginosa* PAO1 (55 °C)^6^, *Thermobifida fusca* KW3 (60 °C)^32^, and *Anoxybacillus* sp. PDF1 (60 °C)^8^. Several studies on thermal stability found that the carboxylesterase of *Bacillus coagulans* 81-11 retained 65% activity after 3 h at 60 °C °C^33^, the carboxylase of *Pseudomonas aeruginosa* PAO1 retained 7% activity after 30 min at 80 °C^6^, and the carboxylesterase of *Thermotoga maritima* retained 50% activity after 30 min at 80 °C^2^. In this study, EstPS1 retained 50% residue activity at 60 °C for 14 h and 50% residue activity at 70 °C for 2 h. Even at 100 °C for 10 min, the enzyme exhibited 19% residue activity. These results indicate that recombinant EstPS1 was more thermotolerant than most of the reported carboxylesterases. A comparison of the thermostability of different sources of carboxylesterases at various temperatures is shown in Table 3. However, the most thermostable carboxylesterase that has been identified is Est from a hyperthermophilic archaeon; the activity of the enzyme did not decrease after 2 h at 100 °C^34^. Est showed higher hydrolytic activity towards esters with short to medium chains, with *p*-nitrophenyl caproate (C6) being the best substrate among the *p*-nitrophenyl esters examined. EstPS1 showed no activity toward *p*-nitrophenyl myristate and *p*-nitrophenyl palmitate. The activity toward the *p*-nitrophenyl esters of medium-chain fatty acids was lower than that toward *p*-nitrophenyl butyrate. Therefore, the *kcat/Km* ratios indicated that the activity of EstPS1 was higher than that of Est towards *p*-nitrophenyl butyrate. The EstPS1 of *P. synxantha* was stable at pH 6.0–9.0 compared with that of *P. aeruginosa* PAO1 (pH 4.0–8.0)^6^, *Geobacillus thermodenitrificans* (pH 6–10)^35^, *Sphingobium* sp. JZ-1 (pH 5.5–9.0)^14^, and *T. fusca* KW3 (pH 5.0–11.0)^32^. The GDSL esterase is active toward short-chain triglycerides (tributyrin) and has low activity towards long-chain triglycerides. EstPS1 showed the highest activity with *p*-nitrophenyl butyrate, and its activity was decreased with increasing aliphatic chain length. Lipolytic activity was very low for esters containing an aliphatic chain length longer than 10 carbon atoms, indicating that EstPS1 is a carboxylesterase and not a lipase. Indeed, lipases prefer substrates with relatively long aliphatic chains. The *Km* value of EstPS1 was 43 μM when *p*-nitrophenyl butyrate was used as a substrate (Table 2). This is similar to other reported carboxylesterases including those from *Pyrobaculum calidifontis* (44 μM)^34^ and *Archaeoglobus fulgidus* (11 μM)^36^. The low *Km* value of EstPS1 makes it an attractive enzyme for industrial applications.

**Table 3:** Comparison of the thermostability of carboxylesterases at different temperatures.

Enzyme	Strain and reference	Optimum	Thermal stability at various temperatures				
		temperature	60 °C	70 °C	80 °C	90 °C	100 °C
EstPS1	*Pseudomonas synxantha* PS1 (this study)	60 °C	50%, 14 h	50%, 2 h	50%, 31 min	50%, 10 min	50%, 1 min
estA	*Pseudomonas citronellolis* ATCC 13674^5^	40 °C	Unstable	—	—	—	—
EstU1	Metagenome^41^	45 °C	Unstable	—	—	—	—
PytH	*Sphingobium* sp. JZ-1^14^	37 °C	55%, 1 h	Inactivated, 1 h	—	—	—
EstC1	*Bacillus coagulans* 81-11^33^	50 °C	65%, 3 h	Unstable	—	—	—
EstGtA2	*Geobacillus thermodenitrificans* CMB-A2^35^	50 °C	93%, 30 min	63%, 10 min	25%, 10 min	—	—
PDF1Est	*Anoxybacillus* sp. PDF1^8^	60 °C	—	50%, 30 min	—	—	—
PAO1	*Pseudomonas aeruginosa* PAO1^6^	55 °C	80%, 2 h	40%, 2 h	7%, 2 h	—	—
EST53	*Thermotoga maritima* tm0053^2^	60 °C	—	78%, 1 h	50%, 30 min	—	—
Est	*Pyrobaculum calidifontis* VA1^34^	90 °C	—	—	—	—	Stable

EstPS1 from *P. synxantha* strain PS1 efficiently hydrolysed the *p*-nitrophenyl esters and degraded all the tested pesticides. Considering that pesticide residues generated from agricultural production are complex mixtures, enzymatic bioremediation requiring the development of specific enzymes for each compound or isomer is unrealistic; thus, pyrethroid-hydrolysing esterases with a broader substrate specificity and higher activity are necessary to fulfil the practical requirements of bioremediation, which would allow the detoxification of pyrethroids where they cause environmental contamination problems^13^. Given that most commercial pyrethroids are mixtures of isomers that may persist in the environment because of isomer-selective degradation, the cloning and overexpression of the pyrethroid-hydrolysing esterase gene can produce enzymes at a low cost for environmental protection. Our results indicate that EstPS1 is an esterase with a broad substrate specificity. This feature is shared by pyrethroid hydrolase from *A. niger* ZD11^28^. A comparison of *Km* and *kcat* revealed that the pyrethroid-hydrolysing EstPS1 has an approximately 6-fold higher affinity toward carbaryl than bifenthrin, and it can hydrolyse the former by approximately 14-fold faster than the latter. Catalytic efficiency values (*kcat*/*Km*) can indicate an enzyme’s specificity; carbaryl and fenpropathrin were clearly the preferred substrates in this study according to catalytic efficiency values. Previous studies have shown that the biodegradation of pyrethroids is commonly isomer-selective. For example, both bacterial and mammalian pyrethroid-hydrolysing carboxylesterases prefer *trans*-permethrin over *cis*-permethrin, whereas the carboxylesterase of *N. cincticeps* Uhler prefers *cis*-permethrin over *trans*-permethrin^27^. Although there have been some reports regarding pyrethroid hydrolase from pyrethroid-resistant insects, mammalian organs, and microorganisms, the pesticide-degrading gene of *P. synxantha* has not been reported.

In conclusion, we identified a novel carboxylesterase (EstPS1) from *P. synxantha*, which has potential for various industrial applications because of its wide pH range and thermostability. The function of the AT domain in enzyme activity and secretion was also assessed. The AT domain as a carrier can be used for both surface display and protein secretion into the extracellular environment. The broad substrate specificity of EstPS1 makes it a suitable candidate for the degradation of pyrethroids in biotechnological applications related to the environment.

## Methods

### Strains, plasmids, and chemicals

*P. synxantha* PS1 was isolated from oil well-produced water (Shengli Oilfield, Shandong Province, China). *E. coli* DH5α (Invitrogen, USA) and plasmid pMD19-T (TaKaRa, Japan) were used for gene cloning and sequencing of AT-containing and AT-truncated carboxylesterases. Plasmid pET-28a (Novagen, USA) was used as a vector to construct the protein expression plasmid in *E. coli* BL21 (DE3). The substrates fenpropathrin, cypermethrin, fenvalerate, bifenthrin, and *p*-nitrophenyl esters were purchased from Sigma (St. Louis, MO, USA). All other chemicals were of analytical grade and purchased from local markets.

### Genomic library construction and screening for thermostable esterase

Genomic DNA from *P. synxantha* PS1 was partially digested by *Pst*I, and the resulting fragments were cloned into the pUC19 vector. The constructed plasmids were transformed into *E. coli* DH5α cells to construct the genomic DNA library. Cells were plated onto LB plates containing ampicillin (50 μg/mL). Transformants were screened on tributyrin agar plates (containing 1.5% tributyrin) and incubated at 37 °C for 24 h to induce enzyme secretion into the surrounding environment. The clones with distinct hydrolysis circles were further screened in a 96-well plate format. Positive strains with high thermostability were isolated, sequenced, and transferred to maintenance slants.

### Carboxylesterase gene and conserved domain analysis

The nucleotide sequence and predicted amino acid sequence were analysed using the BLAST program (NCBI). Carboxylesterase domain analysis was performed by the SMART domain annotation server (http://smart.embl-heidelberg.de/), and the primers (for carboxylesterase gene cloning with/without the AT domain and signal peptide) were designed based on the information obtained from domain analysis. The models were visualised and analysed using PDB Viewer, and the figures were constructed using Pymol.

### Carboxylesterase recombinant strain construction and AT domain expression

To determine the role of the AT domain in the carboxylesterase of *P. synxantha* PS1, four plasmids were constructed. The whole carboxylesterase gene (*Est*PS1) and *Est*PS1 without the AT domain (*Est*PS1**Δ**AT) were cloned using primers F_1_/R_1_ and F_1_/R_2_, respectively (forward primer-F_1_, 5′-CGGATCCATGCTCAAACCAGCGTTTTTC-3′; reverse primer-R_1_, 5′-CCTCGAGTTAGAAATCCAGACTCACCCC-3′; reverse primer-R_2_, 5′-CCTCGAGTTAGGCATAGTCGGCGATCAACTG-3′; underlined nucleotides indicate restriction enzyme sites). *Est*PS1 without the signal peptide (*Est*PS1**Δ**SP) and *Est*PS1 without the signal peptide and AT domain (*Est*PS1**Δ**SP + AT) were cloned using primers F_2_/R_1_ and F_2_/R_2_ (forward primer-F_2_, 5′-CGGATCCACCCCGTCGCCTTATTCAA-3′). PCR was performed in a mixture (50 μL) consisting of Tris-HCl buffer (10 mM, pH 8.0), polymerase buffer (GC buffer), dNTP (0.2 mM), DMSO (2%), and thermostable polymerase LA Taq (1 μL). The amplification protocol was as follows: initial denaturation at 94 °C for 5 min, followed by 30 cycles of denaturation (1 min at 94 °C), annealing (30 s at 55 °C), and extension (90 s at 72 °C), with a final elongation step (10 min at 72 °C). After digestion by *Bam*HI/*Xho*I, the PCR products were recovered and ligated into the pET-28a vector. Recombinant plasmids were transformed into *E. coli* BL21 (DE3) cells. The N-terminal His-tag in the expression vector pET-28a was chosen for all recombinant proteins. The recombinant carboxylesterase strains (named BL21-*Est*PS1, BL21-*Est*PS1**Δ**AT, BL21-*Est*PS1**Δ**SP, and BL21-*Est*PS1**Δ**SP + AT) were maintained for further analysis.

### Effect of AT domain truncation on heterogeneous expression/purification and secretion

The procedures for heterologous protein expression and Ni-NTA purification have been described previously^37^. The crude enzyme was applied to an equilibrated Ni-NTA Superflow column (1 mL, Qiagen) with the lysis buffer (NPI 10: 50 mM NaH_2_PO_4_, 300 mM NaCl, 10 mM imidazole; pH 8.0). The column was subsequently washed with 10 mL of wash buffer (NPI 20: 50 mM NaH_2_PO_4_, 300 mM NaCl, 20 mM imidazole; pH 8.0) to remove impure proteins. Then, the fusion protein was eluted with a linear gradient of washing buffer (from NPI 50 to NPI 250). The eluted protein was desalted and concentrated by ultrafiltration using a 50-mL Amicon Ultra Centrifugal Filter Device with a molecular weight cut-off of 10 kDa (Millipore, USA). The crude extract and the pure enzyme were analysed by SDS-PAGE. All purification steps were carried out at 4 °C. Protein concentration was determined by the Bradford method with bovine serum albumin (BSA) as the standard.

The effect of the AT domain on secretion was investigated using the agar plate hydrolysis circle method and by measuring the secretion rate. For the agar plate hydrolysis circles method, 0.1 μL strain cultures (BL21-*Est*PS1 and BL21-*Est*PS1**Δ**AT) cultured for different periods of time (0, 3, 6, 12, 18, and 24 h) were successively spotted on a tributyrin agar plate (1.5% tributyrin, 50 μg/mL kanamycin, and 0.1 mM IPTG). The plate was incubated at 4 °C to induce enzyme secretion into the surrounding environment. For secretion rate measurement, the enzyme activity of the fermentation supernatant and precipitated cells was detected at different culture times (8, 16, and 24 h), and the secretion rate was calculated based on enzyme activity data. The experiments were repeated 3 times to confirm the reproducibility of the results. Significant differences and variances were evaluated using *Student’s-t* tests. Averaged data were presented as means ± standard deviation (SD). *P* < 0.05 was considered to be significant.

### Analysis of the enzymatic properties of recombinant carboxylesterase

The enzyme activity of purified EstPS1 solution was assayed by measuring the absorbance of liberated *p*-nitrophenol at 405 nm. 1 U is defined as the amount of enzyme releasing 1 μmol *p*-nitrophenol per min under assay conditions. The reaction mixture (0.5 mL) contained 50 μL of *p*-nitrophenyl butyrate solution (final concentration of 25 mM in a solution of isopropanol and dimethyl sulfoxide with a volume ratio of 3:1) as the substrate, 440 μL of lipase assay buffer (50 mM Tris-HCl buffer, pH 8.0), and 10 μL of appropriately diluted enzyme sample. The reaction mixture was incubated at 60 °C for 5 min, cooled in an ice bath, and measured at 405 nm absorbance. Then, enzyme activity was calculated according to the OD and standard curve of *p*-nitrophenol. We assessed substrate specificity using the following substrates: *p*NP-acetate (C2), *p*NP-butyrate (C4), *p*NP-octoate (C8), *p*NP-decenoate (C10), *p*NP-laurate (C12), *p*NP-myristate (C14), *p*NP-palmitate (C16), and *p*NP-stearate (C18).

The optimum pH for enzyme activity was determined over a range of pH (4 to 10) for 5 min at 60 °C. The pH stability of the enzyme was determined by incubating the enzyme in different buffers for 20 h at 30 °C. The following buffer systems were used: 100 mM citric acid-sodium citrate (pH 4.0–5.0), 200 mM sodium phosphate (pH 6.0–7.0), 50 mM Tris-HCl (pH 8.0), and 50 mM glycine-NaOH (pH 9.0–10.0)^37^.

### Optimum temperature and thermal inactivation of recombinant carboxylesterase

Enzyme activity was assayed at 20–80 °C (pH 8.0) to determine the optimum temperature; the reaction mixture containing the appropriately diluted enzyme was incubated at different temperatures for 5 min.

Thermostability is an important factor in enzyme applications. To assess the thermal stability of recombinant EstPS1, the diluted enzyme was incubated for 10 min at various temperatures (60–100 °C, pH 8.0). Aliquots were removed at various intervals, and residual carboxylesterase activity was determined using *p*-nitrophenyl butyrate as the substrate. The sample not incubated at the high temperatures was used as a control.

The half-life of lipase was determined by incubating the enzymes at various temperatures (60–100 °C, pH 8.0) for different time intervals. The enzyme activity at the start of the experiment was taken as 100%. To identify the effect of the AT domain on thermostability, the thermostability and half-life (thermal inactivation T_1/2_) of recombinant EstPS1**Δ**AT were also measured as described above. Each experiment was repeated three times, and each experiment included three replicates. The average values of triplicate measurements were used as each activity value.

### Degradation of pesticides

Increased pyrethroid usage has created a need for green biodegradation, and enzymes have attracted attention as safe and green catalysts. Assays on the pyrethroid-hydrolyzing esterase activity against pesticides were performed as described by Liang *et al*.^38^. Six pesticides (carbaryl, *cis*-cypermethrin, *trans*-cypermethrin, fenpropathrin, fenvalerate, and bifenthrin) were evaluated in this study. EstPS1 (50 U) was added to the pesticide mixture in a final volume of 1 mL, which contained 50 mM Tris-HCl buffer (pH 8.0), the homogenate, and a known concentration (0.5 mM) of pesticides dissolved in dimethyl sulfoxide^39^. The mixture was incubated at 40 °C for 4 h on an orbital shaker (150 rpm). The reaction mixture was saturated by adding NaCl, followed by extraction with 1 mL ethyl acetate^40^. The organic phase was collected and analysed by gas chromatography with a HP-5 column and an electron capture detector. The gas chromatography conditions were as follows: injector temperature of 260 °C, oven temperature of 240 °C, detector temperature of 300 °C, and N_2_ carrier gas at 1 mL/min.

### Determination of kinetic parameters

Kinetic parameters against *p*-nitrophenyl esters were determined by measuring enzyme activity. All measurements were performed in triplicate. The kinetic parameters against different pesticides were analogously determined by measuring the enzyme activity over a range of final concentrations (10 to 500 μM) depending on the different pesticides^20^. All initial velocities were determined at five time points, at which no more than 10% of the substrate had been consumed, and the solution content did not exceed 1% of the total assay volume. Therefore, the decrease in substrate concentration remained linear over the period of measurement, and the rate was relatively consistent throughout the assay. Initial reaction velocities measured at various substrate concentrations were fitted to the Lineweaver-Burk transformation of the Michaelis-Menten equation. All measurements were performed in triplicate.
